# The scaffold protein NEDD9 is necessary for leukemia-cell migration and disease progression in a mouse model of chronic lymphocytic leukemia

**DOI:** 10.1038/s41375-022-01586-1

**Published:** 2022-05-06

**Authors:** Lisa Rusyn, Sebastian Reinartz, Anastasia Nikiforov, Nelly Mikhael, Alexander vom Stein, Viktoria Kohlhas, Johannes Bloehdorn, Stephan Stilgenbauer, Philipp Lohneis, Reinhard Buettner, Sandra Robrecht, Kirsten Fischer, Christian Pallasch, Michael Hallek, Phuong-Hien Nguyen, Tamina Seeger-Nukpezah

**Affiliations:** 1 Faculty of Medicine and Cologne University Hospital, Department I of Internal Medicine, Center for Integrated Oncology Aachen Bonn Cologne Duesseldorf, University of Cologne, Cologne, Germany; 2 CECAD Center of Excellence on Cellular Stress Responses in Aging-Associated Diseases, Center for Molecular Medicine Cologne, Cologne, Germany; 3 Department of Internal Medicine III, Ulm University, Ulm, Germany; 4 Hämatopathologie Lübeck, Reference Centre for Lymphnode Pathology and Haematopathology, Luebeck, Germany; 5 Institute of Pathology, University of Cologne, Cologne, Germany

**Keywords:** Chronic lymphocytic leukaemia, Cell signalling

## Abstract

The scaffold protein NEDD9 is frequently upregulated and hyperphosphorylated in cancers, and is associated with poor clinical outcome. NEDD9 promotes B-cell adhesion, migration and chemotaxis, pivotal processes for malignant development. We show that global or B-cell-specific deletion of Nedd9 in chronic lymphocytic leukemia (CLL) mouse models delayed CLL development, markedly reduced disease burden and resulted in significant survival benefit. NEDD9 was required for efficient CLL cell homing, chemotaxis, migration and adhesion. In CLL patients, peripheral NEDD9 expression was associated with adhesion and migration signatures as well as leukocyte count. Additionally, CLL lymph nodes frequently expressed high NEDD9 levels, with a subset of patients showing NEDD9 expression enriched in the CLL proliferation centers. Blocking activity of prominent NEDD9 effectors, including AURKA and HDAC6, effectively reduced CLL cell migration and chemotaxis. Collectively, our study provides evidence for a functional role of NEDD9 in CLL pathogenesis that involves intrinsic defects in adhesion, migration and homing.

## Introduction

Chronic lymphocytic leukemia (CLL) is characterized by clonal proliferation of mature CD5-positive B-cells that accumulate in the blood and lymphoid organs [1]. Recently, monoclonal antibodies, B-cell receptor (BCR) inhibitors and BCL-2 inhibitors have demonstrated highly promising therapeutic efficacy [1], and impressive results were achieved with their combinations [2, 3]. Despite the undeniable improvement in CLL treatment, resistance to these compounds still pose a challenge in aggressive cases [1, 4].

CLL cells are highly dependent on the tumor microenvironment [5, 6]. Homing into the secondary lymphoid organs and the bone marrow is essential for CLL cells to settle into tumor protective niches and receive pro-survival signals [7]. Massive tissue infiltration of CLL cells leads to splenomegaly and lymphadenopathy and may be responsible for residual disease after therapy [1]. Chemotaxis via chemokine receptors including CXCR4 or CXCR5 regulates CLL cell trafficking via tissue gradients of the their ligands CXCL12 and CXCL13 [8, 9]. Adhesion molecules such as VLA-4 or CD44 mediate homing, adhesion, and retention of CLL cells in lymphoid organs, contributing to disease progression and therapy resistance [7, 10, 11].

The fundamental role of the homing process in CLL progression prompted us to analyze the impact of NEDD9, a crucial regulator of lymphocyte migration, in CLL pathogenesis. NEDD9 (*Neural Precursor Cell Expressed, Developmentally Down-Regulated 9;* also known as Cas-L or HEF1) belongs to the CAS family of non-catalytic scaffolding proteins [12]. Physiologically, NEDD9 is hyperphosphorylated upon activation of cell surface receptors including integrins and the BCR, forming complexes with oncogenic kinases, which subsequently modulate migration, invasion, response to chemokines and cell cycle control [12–15]. Numerous studies indicate that NEDD9 promotes tumor growth and spreading. In tumors, *NEDD9* is rarely mutated but often found overexpressed and constitutively hyperphosphorylated, associated with poor clinical outcome and supposed to boost overreactive oncogenic signaling. However, mice lacking *Nedd9* have no apparent phenotype, despite the near absence of B-cells in the marginal zone, likely due to impaired migration and adhesion capacities [16].

Although NEDD9 was designated to be the lymphocyte-specific member of the CAS family [12], the role of NEDD9 in lymphocytic leukemias has remained unexplored. In this study, we used the well-established *Eµ-TCL1* transgenic mouse model for CLL to decipher the functional relevance of NEDD9 in CLL pathogenesis [17, 18].

## Materials and methods

### CLL samples

Primary CLL cells and lymph node biopsies were obtained from patients after written informed consent according to the Declaration of Helsinki and with Institutional Review Board approval (#11-319 and #13-091) at the University of Cologne. NEDD9 expression was analyzed in 337 patients in the CLL8 trial (NCT00281918) with available peripheral blood samples.

### Mouse experiments

All mouse experiments were approved by the state of North Rhine-Westphalia, Germany #84-02.04.2014.A146, #81-02.04.2019.A009, and #84-02.04.2016.A058. Husbandry, procedures for blood and organ sample collection, and differential blood counts were described previously [19]. The *Nedd9* conditional knockout mouse model (*Nedd9*^*fl/fl*^) was generated under contract with Cyagen Biosciences Inc. (Santa Clara, CA, USA) (Supplementary Data).

### In vivo homing assay

Splenic cells of *TCL1*^*tg/wt*^*Nedd*^*wt/wt*^ or *TCL1*^*tg/wt*^*Nedd9*^*−/*−^ mice were labelled with CFSE (Abcam) and injected into the tail vein of 8-week-old recipient mice. The inoculum consists of an absolute number of 10^6^ CD5^+^CD19^+^ CLL cells based on flow cytometric analysis. CFSE^+^ cells were detected in spleen and bone marrow of the recipients 3 h after injection.

### Migration assay

MEC1, murine- or human CLL cells (10^6^) were serum-starved for 2 h, then seeded in 6.5 mm trans-wells with 5 µm pore size (Corning). In indicated experiments cells were treated with 0.5 µM alisertib or 0.5 µM panobinostat for 2 h. The lower compartment contained 10% FCS, or 200 ng/µl CXCL12, or RPMI only. Number of migrated cells was determined after 4 h by FACSCalibur™ for 60 s on high flow rate. Migration index was calculated as the ratio of spontaneous migration (no stimulus) to stimulated migration (FBS or CXCL12).

### Adhesion assays to fibronectin and bone marrow stroma cells

CLL cells (5 × 10^6^) were plated on uncoated or 2.5 µg/ml fibronectin-coated 12 mm coverslips for 1 h. After repeated washing steps, coverslips with attached cells were fixed with ice-cold methanol and stained with Hoechst. Attached cells were counted at 20x magnification in at least 10 fields of view for each cover slip. Bone marrow stroma cells (BMSC, 5 × 10^5^/well) were flushed from femurs and tibias of wildtype mice. CLL cells (5 × 10^5^) were labeled with 5 µM CellTracker™ Red CMTPX Dye (Thermo Fischer Scientific) and co-cultured with BMSC for 24 h. Adhered CLL cells were scraped off and counted by Gallios cytometer for 60 s on high flow rate.

### Microarray data

Sampling was performed before initiation of treatment for treatment naïve patients (*n* = 337) [20]. mRNA extraction, quality control and the Affymetrix GeneChip^®^ Human Exon 1.0 ST Array (Affymetrix, Santa Clara, CA, USA) for expression profiling were previously described [21].

### Statistical analysis

Statistical analysis was performed with Prism 9.0.0 (GraphPad Software, La Jolla, CA). Data were presented as median, non-parametric Mann-Whitney tests were performed to analyze the significance, if not otherwise indicated. Overall survival was analyzed with Log-rank test. Multiple comparisons were analyzed by Sidak’s multiple comparison test. *P* > 0.05 equals not significant (ns), *p ≤ 0.05, **p ≤ 0.01, ***p ≤ 0.001 and ****p ≤ 0.0001.

## Results

### Nedd9 depletion impairs CLL cell infiltration and prolongs survival in the *Eµ-TCL1* mouse model

**Fig. 1:**
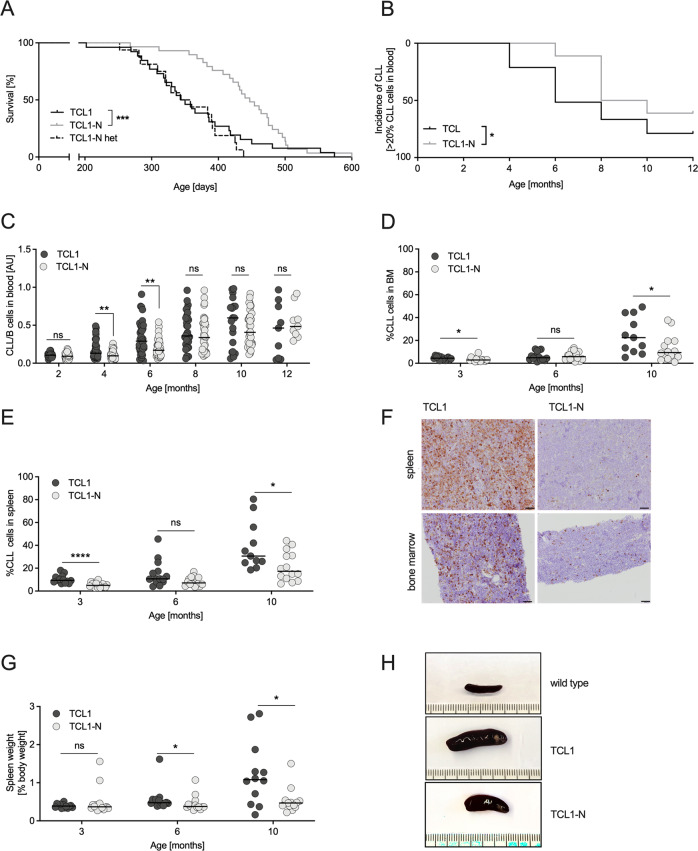
Nedd9 depletion impairs CLL cell infiltration and prolongs survival in the *Eµ-TCL1* mouse model. **A** Kaplan–Meier curve representing the overall survival of *Nedd9* knockout (TCL1-N), *Nedd9* heterozygous (TCL1-N het) and *Nedd9* wildtype (TCL1) TCL1 mice from birth to moribund. Median survival TCL1 = 347 days (*n* = 26), TCL1-N het = 350.5 days (*n* = 16), TCL1-N = 447 days (*n* = 29), *p* = 0.0003. **B** Flow cytometric analysis of CD19^+^CD5^+^ CLL cells in peripheral blood mononuclear cells (PBMC) of TCL1 and TCL1-N mice at indicated time points presenting CLL incidence, defined as CLL cells in PBMC > 20%. *P* = 0.01. **C** Flow cytometric analysis of CD19^+^CD5^+^ CLL cells in PBMC of TCL1 and TCL1-N mice at indicated time points (as ratio CLL/B-cells). *P* (2 months) = 0.2884; *p* (4 months) = 0.0087; *p* (6 months) = 0.0026; *p* (8 months) = 0.5044; *p* (10 months) = 0.4241; *p* (12 months) = 0.4894. **D** Flow cytometric analysis of CD19^+^CD5^+^ CLL cells in bone marrow samples of age-matched TCL1 and TCL1-N mice. *P* (3 months) = 0.0129; *p* (6 months) = 0.7345; *p* (10 months) = 0.0442. **E** Flow cytometric analysis of CD19^+^CD5^+^ CLL cells in spleens of age-matched TCL1 and TCL1-N mice. *P* (3 months) < 0.0001; *p* (6 months) = 0.11; *p* (10 months) = 0.04. **F** Representative immunohistochemical stainings of CD45R from sections of spleen and bone marrow from ten months old TCL1 and TCL1-N mice. Scale bars represent 50 μm. **G** Spleen weight relative to body weight of age-matched TCL1 and TCL1-N mice. *P* (3 months) = 0.59; *p* (6 months) = 0.01; *p* (10 months) = 0.02. **H** Representative pictures of spleens from ten months old wildtype, TC and TCN mice. Ruler unit in mm/cm.

Using the *Eµ-TCL1* mouse model [17, 18], we generated *TCL1*^*tg/wt*^*Nedd9*^*wt/wt*^ and *TCL1*^*tg/wt*^*Nedd9*^*−/−*^ [16] cohorts (designated TCL1 and TCL1-N mice, respectively) to analyze the consequence of constitutive Nedd9 depletion for CLL pathogenesis. Intriguingly, the overall survival was vastly prolonged in the TCL1-N group (*n* = 29) compared to the TCL1 group (*n* = 26) with median survival of 447 days and 347 days, respectively (*p* = 0.0003). A TCL1 cohort with heterozygous Nedd9 (*TCL1*^*tg/wt*^*Nedd9*^*wt/−*^ *n* = 16) showed a median survival of 350.5 days (Fig. 1A, S1A). This survival difference reflects the fact that loss of Nedd9 significantly delayed CLL onset. While 21.21% of the TCL1 group showed CLL burden (defined by >20% CLL cells in peripheral blood mononuclear cells; PBMC) by month 4, CLL was not detected in the TCL1-N group until month 6, with a proportion of only 11.12% (Fig. 1B). The early reduction of CLL cells in the blood of TCL1-N versus TCL mice was lost at later time points, which reflects the fact that the TCL1 animals with advanced CLL succumbed faster than TCL1-N animals, and thus were no longer represented in the analysis (Fig. 1C).

In accord with the differences in PBMCs, CLL infiltration in bone marrow and spleen was clearly reduced in TCL1-N mice compared to TCL1 counterpart, with the largest differences visible with advanced disease at month10 (Fig. 1D, E). This was reflected in the immunohistochemical staining of CD45R^+^ B-cells heavily infiltrating spleens and bone marrows of TCL1 mice in contrast to limited infiltration in TCL1-N mice (Fig. 1F). This was accompanied by TCL1-N mice showing relatively normal sized spleens even at month 10 compared to TCL1 mice (Fig. 1G, H), which exhibited significantly enlarged spleens already at month 6 (Fig. 1G). Additionally, in TCL1 mice CLL cell expansion in the bone marrow suppressed normal hematopoiesis, resulting in low blood platelets and erythrocyte levels, whereas TCL1-N mice showed more stable thrombocyte and erythrocyte counts (Fig. S1B, C).

Taken together, loss of Nedd9 remarkably delayed CLL onset and progression in vivo, particularly impaired CLL cell infiltration in lymphoid organs, leading to a substantial survival advantage in mice.

### B-cell-specific loss of Nedd9 is sufficient to reduce CLL burden

**Fig. 2:**
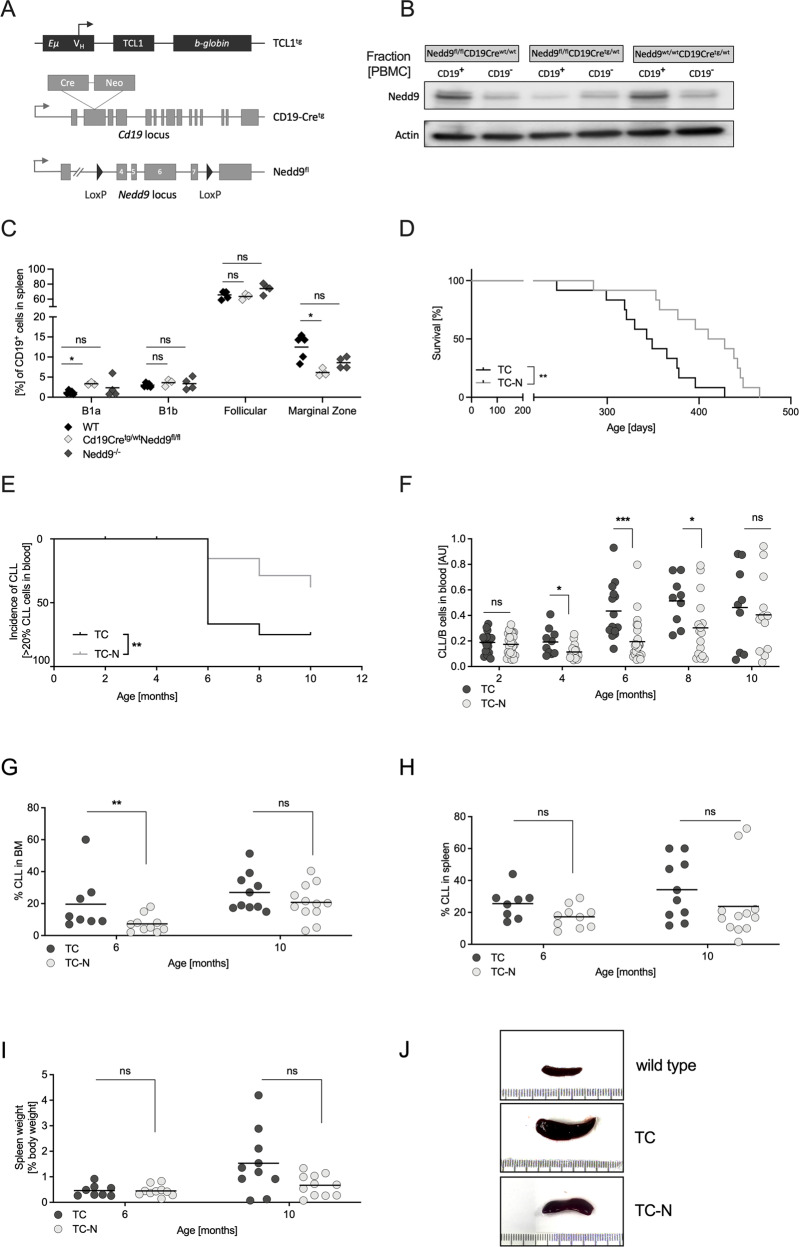
B-cell specific loss of Nedd9 is sufficient to reduce CLL burden in TCL1 mice. **A** Schematic representation of novel genetic loci for B-cell specific Nedd9 knockout. **B** Confirmation of B-cell specific loss of Nedd9 protein upon *Cd19*-dependent Cre-recombination. Cd19^+^ and Cd19^−^ cells were sorted from PBMC of mice with indicated genotypes using mouse Cd19-specific magnetic beads and indicated protein lysates where blotted for Nedd9. **C** Flow cytometric analysis of different B-cell subsets in spleens of WT (*n* = 4), *CD19Cre*^*tg/wt*^*Nedd9*^*fl/fl*^ and *Nedd9*^*−/−*^ mice, *p* (B1a cells *CD19Cre*^*tg/wt*^*Nedd9*^*fl/fl*^ vs. WT) = 0.035714; *p* (marginal zone B-cells *CD19Cre*^*tg/wt*^*Nedd9*^*fl/fl*^ vs. WT) = 0.035714. **D** Kaplan–Meier curve representing the overall survival of B lineage-specific Nedd9 knockout (TC-N), and control cohort (TC) from birth to moribund. Median survival TC-N = 419 days (*n* = 12), TC = 346 days (*n* = 12), *p* = 0.0031. **E** Flow cytometric analysis of CD19^+^CD5^+^ CLL cells in the peripheral blood of mice with (TC-N) and without (TC) B lineage-specific Nedd9 loss at indicated time points (as ratio of CLL cells/B-cells). *P* (2 months) = 0.6355; *p* (4 months) = 0.0273; *p* (6 months) = 0.0001; *p* (8 months) = 0.0369; *p* (10 months) = 0.6016. **F** Flow cytometric analysis of CD19^+^CD5^+^ CLL cells in PBMC of TC and TC-N mice at indicated time points presenting CLL incidence (CLL cells in PBMC > 20%). *P* = 0.0039. **G** Flow cytometric analysis of CD19^+^CD5^+^ CLL cells in bone marrow samples of age-matched TC and TC-N mice. *P* (6 months) = 0.0.0050; *p* (10 months) = 0.4664. **H** Flow cytometric analysis of CD19^+^CD5^+^ CLL cells in spleens of age-matched TC and TC-N mice. *P* (6 months) = 0.0782; *p* (10 months) = 0.0513. **I** Spleen weight relative to body weight of age-matched TC and TC-N mice. *P* (6 months) > 0.9999; *p* (10 months) = 0.0845. **J** Representative pictures of spleens from ten months old wildtype, TC and TC-N mice. Ruler unit in mm/cm.

To examine if B-cell-specific loss of Nedd9 contributes to CLL pathogenesis, we generated a conditional mouse model with loss of Nedd9 exclusively in the B-cell lineage (*CD19Cre*^*tg/wt*^*Nedd9*^*fl/fl*^). We crossed those mice to TCL1 mice generating cohorts of CLL mice with and without Nedd9 loss in the B-cell compartment, designated TC-N (*TCL1*^*tg/wt*^*CD19Cre*^*tg/wt*^*Nedd9*^*flfl*^) and TC mice (*TCL1*^*tg/wt*^*CD19Cre*^*wt/wt*^*Nedd9*^*flfl*^), respectively (Fig. 2A, B). The newly generated *CD19Cre*^*tg/wt*^*Nedd9*^*fl/fl*^ mice did not show any detectable differences in the most abundant immune cell types in spleen and bone marrow compared with WT and *Nedd9*^*−/−*^ mice (Fig. S1D, E). Concerning the different splenic B-cell subsets (Fig. S1F), *CD19Cre*^*tg/wt*^*Nedd9*^*fl/fl*^ mice exhibited a similar reduction of marginal zone B-cells as described for *Nedd9*^*−/−*^ mice [16] (Fig. 2C). Notably, the CD5^+^ B1a-B-cell subset that is considered the normal counterpart of CLL cells and could give rise to CLL cells was increased in *CD19Cre*^*tg/wt*^*Nedd9*^*fl/fl*^ mice [22]. Indeed, the phenotype of TC-N mice highly resembled that of TCL1-N mice, with overall survival prolonged in the TC-N group (*n* = 12; 419 days) compared to the TC group (*n* = 12; 346 days) (*p* = 0.0031) (Fig. 2D). CLL onset was substantially delayed in TC-N compared to TC mice (Fig. 2E), and there were significantly fewer CLL/B-cells in the peripheral blood of TC-N mice at months 4, 6 and 8 than of TC mice (Fig. 2F). Likewise, B lineage-specific Nedd9 loss impaired CLL cell infiltration into the bone marrow and spleen (Fig. 2G, H), with the latter reflected by a trend towards reduced spleen weight (Fig. 2I, J). In summary, we show that B-cell-specific Nedd9 loss was sufficient to significantly delay CLL onset and progression, and to decrease the accumulation of CLL cells both in blood and homing organs. This confirms the functional role of Nedd9 in CLL pathogenesis in a second independent mouse model, and highlights B-cell intrinsic activity of Nedd9 as the major contributor to this effect.

### Nedd9 loss impairs homing of CLL cells to lymphoid organs

**Fig. 3:**
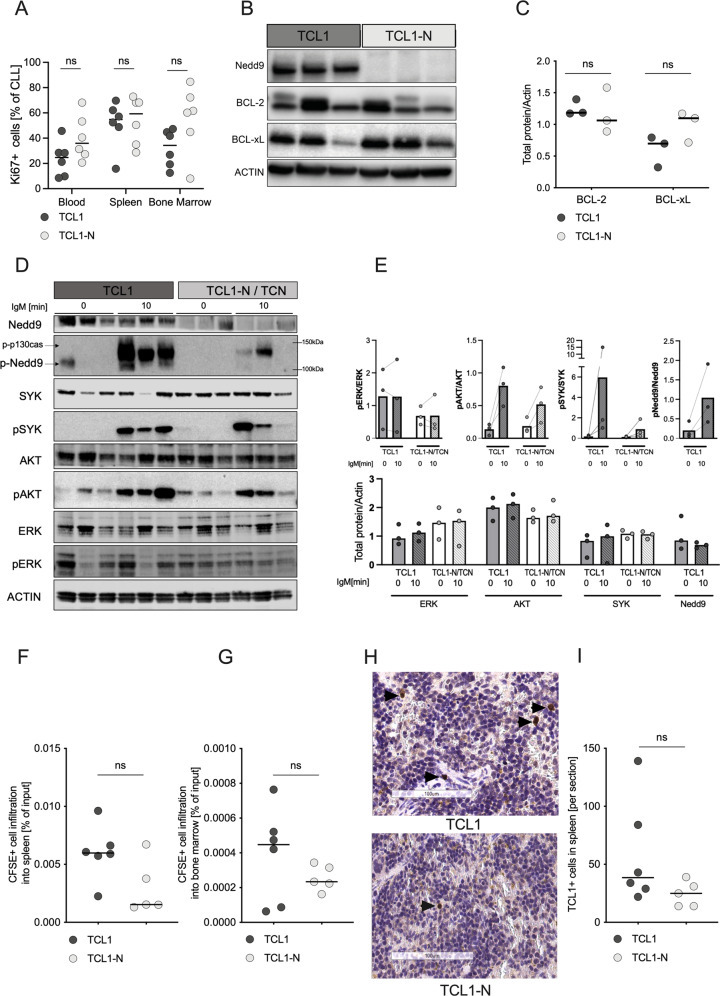
Nedd9 loss impairs homing of CLL cells to lymphoid organs. **A** Flow cytometric analysis of Ki67^+^ CLL cells indicating proliferation in blood, spleen and bone marrow samples of ten months old TCL1 and TCL1-N mice. *P* (blood) = 0.1797; *p* (spleen) = 0.8182; *p* (bone marrow) = 0.0931. **B** CLL cells isolated from moribund TCL1 or TCL1-N mice and examined for protein levels of Nedd9, BCL-2, BCL-xL and β-Actin by Western Blot. **C** Western blot quantification of BCL-2 and BCL-xL by lab image 1D, normalization on mean of all samples. *N* = 3; *p* (BCL-2) = 0.7000; *p* (BCL-xL) = 0.2000. **D** CLL cells isolated from individual moribund TCL1 or TCL-N mice. Cells from each individual mouse were kept untreated or stimulated with 20 µg/ml IgM for the indicated time before protein lysis and Western Blot analysis of indicated candidates. Phosphorylated Nedd9 was detected with an antibody specific for phosphorylated p130Cas [48] that detects phosphorylated Nedd9 between 100–115 kDa. **E** Western blot quantification of pERK, pAKT, pSYK, pNedd9 normalized to total respective protein, and total protein levels normalized to Actin by lab image 1D. **F** Flow cytometric analysis of CFSE^+^ CLL cells of TCL1 and TCL1-N mice homing to the spleen and (**G**) bone marrow of NOD/SCID mice three hours post intravenous injection. *P* (spleen) = 0.1255); *p* (bone marrow) = 0.4286. **H** Representative immunohistochemical staining with specific antibody to human TCL1 of sections from spleens of NSG recipients after in vivo homing assays with TCL1 and TCL1-N cells. **I** Quantification of TCL1 staining spleens of recipient TCL1 and TCL1-N mice *P* = 0.1190. Histology slides were scanned and analyzed with the Aperio ImageScope – Pathology Slide Viewing Software (Leica, Wetzlar, Germany). Six fields per slide were analyzed at 20x magnification.

To understand the basis of the considerable impact of Nedd9 loss on CLL development, we assessed whether Nedd9 loss reduced CLL cell proliferation and survival signaling. CLL cells of both TCL1-N and TC-N mice revealed proliferation rates comparable to age-matched controls, based on Ki67 expression in blood and tissues (Fig. 3A, S2A). Expression of the major regulators of CLL survival, BCL-2 and BCL-xL showed varying levels but did not significantly differ between TCL1 and TCL1*-*N CLL cells (Fig. 3B, C, S2B). Although Nedd9 phosphorylation was induced in the TCL1 CLL cell within 10 min of BCR stimulation, *Nedd9* deletion did not affect either baseline or anti-IgM-induced expression and phosphorylation of BCR downstream targets such as ERK1/2 and AKT (Fig. 3D, E), and could be confirmed by longer-time dynamics of BCR stimulation in additional pools of CLL cells (Fig. S2D). These data do not suggest a major impact of NEDD9 on B-cell-intrinsic proliferation, apoptosis or BCR signaling pathway.

Instead, the significant delay of leukemic infiltration into lymphoid organs upon Nedd9 loss implied that Nedd9 depletion might alter CLL cell homing. Therefore, we conducted a short-term homing assay with TCL1 and TCL1-N CLL cells. CFSE-labeled CLL cells from each donor was injected intravenously into recipient mice 3 h prior to organ collection for infiltration analysis. In immune competent C57B/l6-J recipients, CFSE-positive TCL1-N CLL cells (mixed C57B/l6-J/N) had the tendency to infiltrate less into recipients’ spleens compared to TCL1 cells (Fig. S2C, D), although there was no difference in bone marrow infiltration (Fig. S2C). When NOD-*scid;*IL2Rg^null^ (NSG) mice were used as recipients to exclude the influence of immune defense on tumor cells, CSFE-positive TCL1-N cells also showed a distinct trend of less effective homing to spleens compared to TCL1 cells (Fig. 3F, G). For confirmation, we stained sections of NSG recipients’ spleens for TCL1-expressing B-cells, which again showed reduced capacity of Nedd9-depleted CLL cells to infiltrate (Fig. 3H, I). These data indicated Nedd9 enabled efficient CLL cell homing to lymphoid tissues, an important mechanism to promote CLL progression.

### Loss of Nedd9 impairs multiple steps of the CLL homing process, including adhesion and migration

**Fig. 4:**
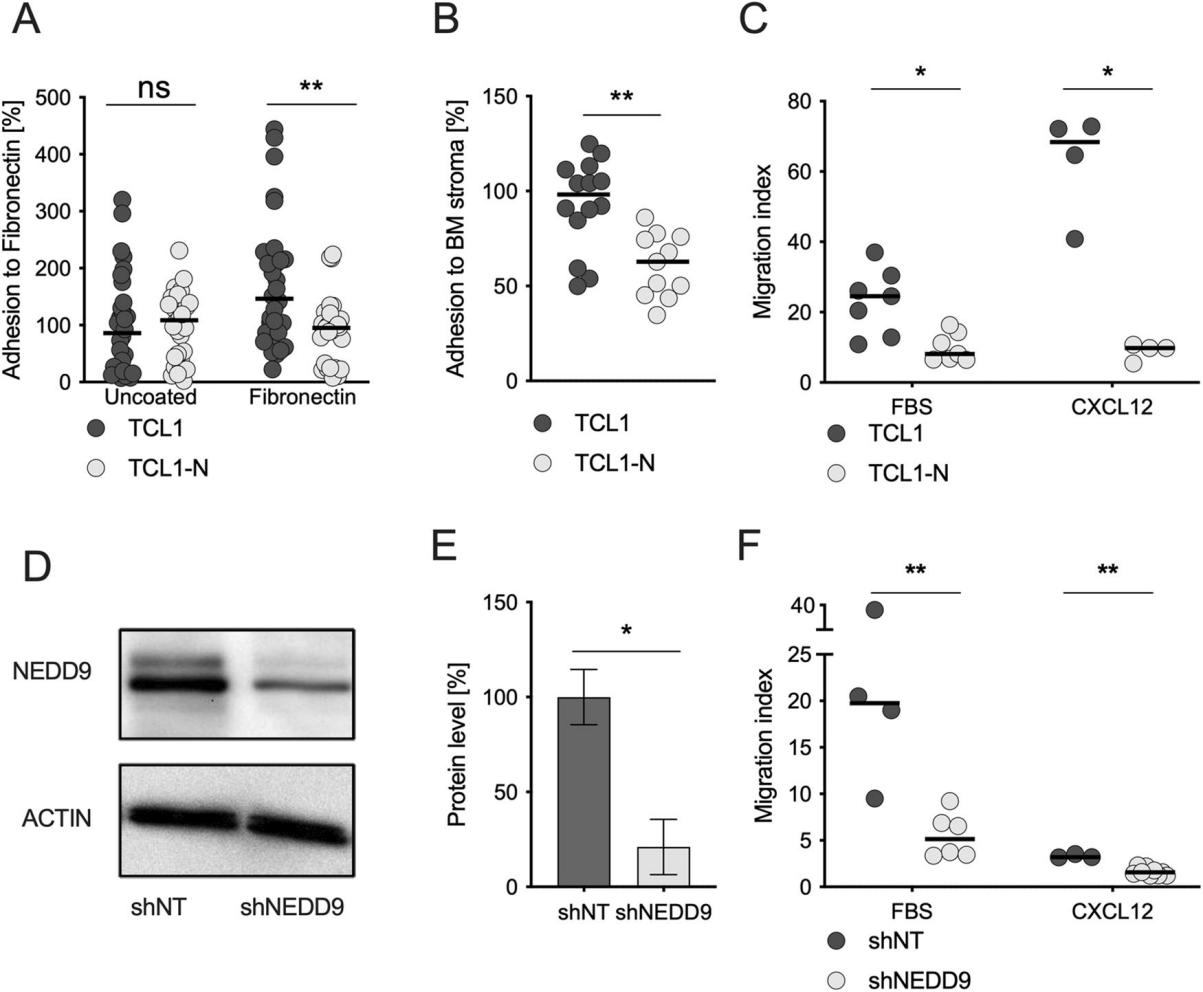
Loss of Nedd9 impairs multiple steps of the CLL homing process, including adhesion and migration. **A** CLL cells isolated from moribund TCL1 or TCL1-N mice were analyzed for cell adhesion after 60 min to uncoated or fibronectin (FN) coated cover slips and plotted as % of TCL1 CLL cell adhesion to uncoated cover slips. *P* (uncoated) = 0.5133; *p* (Fibronectin) = 0.0061. **B** Primary murine CLL cells were allowed to adhere to bone marrow cells derived from wildtype mice for 24 h followed by FACS analysis of adherent cells. *P* = 0.0014. **C** Primary murine CLL cells isolated from moribund mice were analyzed for migration capacity towards FBS or 200 ng/µl CXCL12 or control (serum free media) using a Boyden chamber. Each biological sample were measured in duplicate, each dot represents the mean of the duplicates. Migration index was calculated as the ratio of spontaneous migration to stimulated-migration. *P* (FBS) = 0.0111; *p* (CXCL12) = 0.02686. **D** MEC1 cells were transfected with lentiviral vectors either expressing non-target shRNA (shNT) or shRNA directed against NEDD9 (shNEDD9). Representative western blotting image is shown. **E** NEDD9 protein levels were quantified using lab image 1D software (Kaplan Bio-Imaging GmbH, Leipzig, Germany), normalization to mean β-actin. *N* = 4, *p* = 0.0286. Data are presented as mean ± SD. **F** MEC1 cells transfected with shNT (one single cell clone) and MEC1 cells transfected with shNEDD9 (two different single cell clones) were examined for migration capacity towards FBS, 200 ng/µl CXCL12 or control (serum free media) using a Boyden chamber. Migration index was calculated as the ratio of spontaneous migration (control, no stimulus) to directed migration (stimulated cells, FBS or CXCL12). *p* (FBS) = 0.0095; *n* (shNT) = 4; *n* (shNEDD9) = 6; *p* (CXCL12) = 0.0091; *n* (shNT) = 3; *n* (shNEDD9) = 9.

To test the effect of Nedd9 loss on adhesion and migration that are essential processes of lymphoid homing, we performed an adhesion assay with fibronectin (FN), a major adhesion molecule in CLL homing [23]. TCL1-N CLL cells completely failed to adhere to FN, whereas TCL1 CLL cells showed a major increase in adhesion to FN-coated plates compared to uncoated ones (Fig. 4A). TCL1-N CLL cells also had significantly impaired adhesion to BMSC, compared to the strong adhesion by TCL1 cells (Fig. 4B). Additionally, loss of Nedd9 markedly decreased CLL cell migration in transwell assays, reducing both migration of CLL cells towards FBS as a mixture of chemoattractants (Fig. 4C, left), and directed chemotaxis towards CXCL12 that induced massive migration of CLL cells (Fig. 4C, right) [8, 24].

These results from murine models were confirmed in the human CLL-like cell line MEC1. MEC1 cells were stably transfected with shRNA targeting *NEDD9* (shNEDD9), showing a 80% reduction of NEDD9 protein level compared to non-targeted controls (shNT) (Fig. 4D, E). Similar to the murine system, NEDD9-deficient MEC1 cells were vastly impaired in their migration capacity towards FBS (Fig. 4F, left). Although MEC1 cells migrated much less efficiently towards CXCL12 compared to FBS due to low level of CXCR4 (Fig. S3A) [25], NEDD9-knockdown was sufficient to abrogate the remaining migration capacity towards CXCL12 compared to shNT cells (Fig. 4F, right). NEDD9 depletion in MEC1 cells did not change cell intrinsic proliferation or apoptosis rates (Fig. S3B, C). Altogether, the primary result of NEDD9 deficiency was to impair important steps of CLL cell homing by disrupting adhesion and chemotaxis.

### NEDD9 expression is associated with adhesion and migration signatures in CLL cells and frequently accentuated in proliferation centers of the CLL lymph nodes

**Fig. 5:**
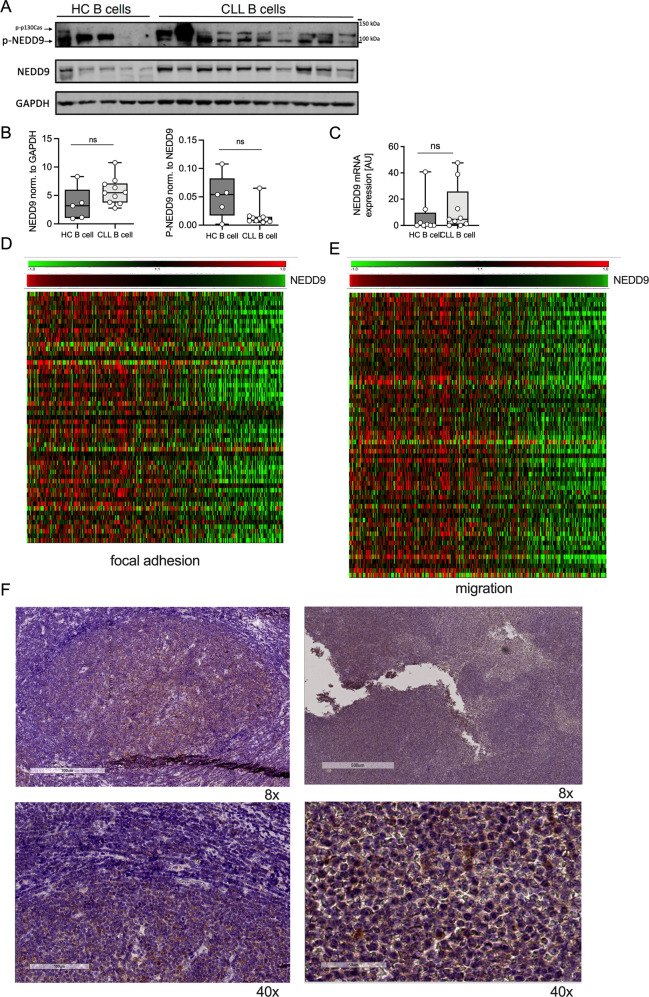
NEDD9 expression is associated with adhesion and migration signatures in CLL cells and frequently accentuated in proliferation centers of the CLL lymph nodes. **A** B lymphocytes were isolated from PBMC of 10 CLL patients (CLL B-cells) and 5 healthy control donors (HC B-cells). Phospho-NEDD9 and NEDD9 levels were analyzed by Western blotting. Phosphorylated Nedd9 was detected with an antibody specific for phosphorylated p130Cas [48] that detects phosphorylated NEDD9 between 100–115 kDa. **B** Western blot quantification of phospho-NEDD9 and NEDD9 levels between healthy controls and CLL samples. *p* (NEDD9/GAPDH) = 0.1292; *p* (pNEDD9/NEDD9) = 0.1645. **C** B lymphocytes were isolated from PBMC from CLL patients and healthy control (HC) donors and *NEDD9* mRNA level were analyzed using real-time PCR. *N* ≥ 7, *p* = 0.5358. **D**, **E** Microarray analysis of NEDD9 expression levels of CD19-sorted primary CLL samples (*n* = 337) and association with genes involved in (**D**) focal adhesions and (**E**) migration. **F** Representative immunohistochemical stainings of NEDD9 of lymph node section from CLL patients. Magnification 8x and 40x.

To establish clinical relevance of NEDD9, we investigated phospho- and total NEDD9 expression levels in purified leukemic cells from the peripheral blood of CLL patients versus sex- and age-matched healthy donors (Fig. 5A, S4A). When comparing phospho-NEDD9 levels normalized to total NEDD9 levels, we noticed a great variance in healthy B-cells either showing strong NEDD9 phosphorylation or almost none, which is typical for a dynamic regulation of NEDD9 expression under physiological conditions. In contrast, phospho-NEDD9 levels in CLL cells were much more consistent among patient samples, displaying a minimal variance of consistently low NEDD9 phosphorylation. Due to the wide range in healthy B-cells the results showed no significance in the mere phosphorylation strength (Fig. 5B). In line with the rigid NEDD9 activation in CLL cells, we observed a lack of NEDD9 mRNA induction in response to BCR stimulation in a previous microarray screen in CLL cells versus healthy controls that is independent of the IGVH mutational status (Fig. S4B, C). However, baseline levels of total NEDD9 protein or *NEDD9* mRNA showed comparable expression patterns among healthy B-cells and CLL cells (Fig. 5A, C). Altogether, these results indicate a constant activation of NEDD9 in CLL cells with loss of responsiveness to cellular stimuli such as BCR activation.

Given the role of Nedd9 in murine cell adhesion and migration, we correlated NEDD9 expression levels in a microarray with expression patterns of genes involved in these processes in patients in the CLL8 trial of the German CLL Study Group. Here, a clear association between NEDD9 transcriptional levels and various adhesion and migration genes could be observed (Fig. 5D, E; gene names indicated in Fig. S4D, E). We therefore explored if NEDD9 is particularly expressed within the lymphatic tissue - the destination of CLL cell homing, and examined NEDD9 expression in CLL lymph nodes (*n* = 21) and healthy controls (*n* = 3). CLL lymph nodes characteristically show proliferation centers comprising CLL cells which are associated with increased cytogenetic aberrations, aberrant expression of oncogene and tumor-suppressor microRNAs, and aggressive clinical behavior [26, 27]. NEDD9 expression in CLL lymph nodes was accentuated in proliferation centers for a subgroup of CLL patients (33%, *n* = 7); for 38% of patients, there was a diffuse pattern of NEDD9 expression in CLL infiltrating cells (*n* = 8), while there was negative staining in 28% (*n* = 6) of the cases (Fig. 5F, Table S1). Healthy control samples showed NEDD9 expression predominantly in the germinal centers of the secondary lymphoid organ follicles (Fig. S4F), where B-cell proliferation and maturation takes place. Taken together, *NEDD9* mRNA expression in primary CLL cells from peripheral blood is highly associated with adhesion and migration signaling, and in human CLL lymph nodes NEDD9 protein expression is found frequently, either as diffuse pattern or accentuated in proliferation centers. Although we could not find a correlation between NEDD9 expression in peripheral CLL cells and bulky disease of CLL8 patients (Table 1), we discovered a strong positive correlation between NEDD9 levels (above/below median) and leukocyte count – a hallmark of CLL aggressiveness (Table 1), further implying a functional role for NEDD9 in CLL pathogenesis.

**Table 1:** Association of continuous NEDD9 expression with clinical features at baseline in CLL8 cohort.

Patient characteristic at baseline	Categories	*N*	Median NEDD9 expression	*P* value
All patients		337	6.421	
Leukocyte count ≥ 50.0 × 10^9^/L	No	83	6.263	**<0.001**
	Yes	247	6.469	
Lymph nodes > 5 cm	No	140	6.367	0.952
	Yes	38	6.359	
Lymph nodes > 10 cm	No	166	6.370	0.483
	Yes	12	6.327	
Splenomegaly > 15 cm	No	58	6.343	0.204
	Yes	45	6.497	
Splenomegaly > 20 cm	No	90	6.359	0.275
	Yes	13	6.505	

Correlations between continuous NEDD9 expression and leukocyte count, enlargement of lymph nodes, and splenomegaly at baseline time point were evaluated using Mann–Whitney *U* test. The analyses of lymph node and spleen sizes refer only to patients with measurements via CT scan. A p-value of <0.05 was defined as significant (bold value). No adjustments for multiple testing were done. The analyses were performed using SPSS 27.0.

### Blocking effector molecules of the NEDD9 signaling network partially reduces migration capacity of CLL cells

**Fig. 6:**
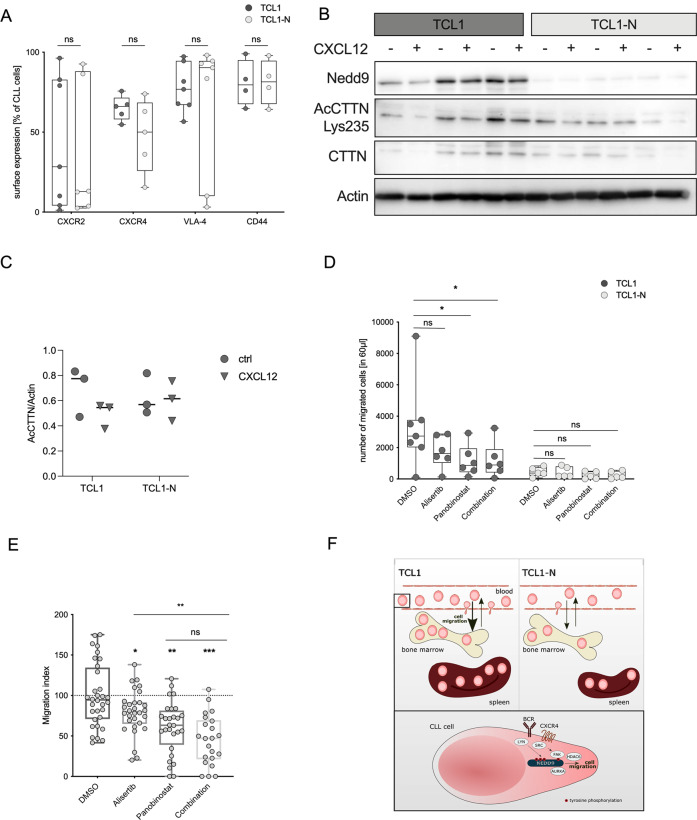
Blocking effector molecules of the NEDD9 signaling network partially reduces migration capacity of CLL cells. **A** Primary CLL cells from ten months old TCL1 and TCL1-N mice were examined for surface expression of indicated adhesion molecules by flow cytometry and plotted by percentage of CLL cells. *N* = 6; *p* (CXCR2) = 0.9452; *p* (CXCR4) = 0.2222; *p* (VLA-4) = 0.8048; *p* (CD44) > 0.999. **B** Primary CLL cells isolated from moribund TCL1 and TCL1-N mice were stimulated with 200 ng/µl CXCL12 for 15 min followed by western blot analysis and (**C**) quantitative analysis. **D** Primary CLL cells isolated from moribund TCL1 and TCL1-N mice were treated with 0.5 µM alisertib, 0.5 µM panobinostat or a combination of both and examined for migration capacity towards 200 ng/µl CXCL12 using a Boyden chamber. For TCL1 cells, *p* (DMSO vs. alisertib) = 0.0781; *p* (DMSO vs. panobinostat) = 0.0312; *p* (DMSO vs. combination) = 0.0156. For TCL1-N cells, *p* (DMSO vs. alisertib) = 0.8125; *p* (DMSO vs. panobinostat) = 0.0625; *p* (DMSO vs. combination) = 0.0625. Statistics by Wilcoxon matched pairs test. **E** Patient-derived primary CLL cells were treated with 0.5 µM alisertib (*n* = 30), 0.5 µM panobinostat (*n* = 28) or a combination of both (*n* = 22) and examined for migration capacity towards 200 ng/µl CXCL12 using a Boyden chamber. *P* (DMSO vs. alisertib) = 0.00234; *p* (DMSO vs. panobinostat) = 0.0024; *p* (DMSO vs. combination) = 0.0002, *p* (alisertib vs. combination = 0.0023), *p* (panobinostat vs. combination) = 0.4749. Statistics by Wilcoxon matched pairs test. **F** Schematic representation of proposed NEDD9-dependent signaling axis.

NEDD9 may influence CLL homing and chemotaxis by causing changes in surface expression of the prominent cell trafficking mediators CXCR2, CD44, VLA-4, and CXCR4. However, we did not detect differences in expression of these proteins between TCL1 and TCL1-N cells (Fig. 6A). Therefore, we considered that impaired chemotaxis to CXCL12 in Nedd9-knockout cells is a consequence of defects in the intracellular signal transduction network. NEDD9 is well described to co-activate the signaling axis AURKA/HDAC6 to induce cytoskeletal changes [28], and AURKA and HDAC6 were independently shown to promote CXCL12 dependent migration [29]. Moreover, Cortactin (CTTN) – another relevant intermediate molecule of the actin cytoskeleton machinery which is overexpressed in CLL patients [30] – also promotes CLL cell migration towards CXCL12, and was described as a major mediator of NEDD9-driven migration [31]. CTTN complexes with NEDD9 where its deacetylation is fostered by NEDD9-dependent AURKA/HDAC6 activity [28]. Indeed, we observed a tendency of reduced level of acetylated cortactin (AcCTTN) upon CXCL12 treatment in murine CLL cells with intact NEDD9 (Fig. 6B, C, left). In contrast, CXCL12 did not influence AcCTTN in TCL1-N cells (Fig. 6B, C, right), suggesting defective AURKA/HDAC6 activity in the knockout cells. Furthermore, TCL1-N cells expressed slightly lower levels of CTTN than TCL1 cells. As specific agents blocking NEDD9 or CTTN are still lacking, we tested whether targeting the effector kinases AURKA and HDAC6 could mimic NEDD9 depletion effects. We analyzed the potential of AURKA inhibitor alisertib and HDAC inhibitor panobinostat for blocking TCL1 and TCL1*-*N CLL cell migration. Alisertib showed a clear trend to slow down migration and panobinostat either alone or in combination with alisertib significantly impaired migration of murine CLL cells towards CXCL12. Importantly, this activity was dependent on the presence of Nedd9 because drug effect was significantly diminished in TCL1*-*N cells (Fig. 6D). In agreement with the murine models, both alisertib and panobinostat could reduce patient-derived CLL cell chemotaxis towards CXCL12 significantly (Fig. 6E), with the combination treatment showing additive effects. Particularly, the effectiveness of alisertib and panobinostat to reduce migration could be observed across different CLL subgroups (Fig. S6), including mutated IgHV (blue, 3/3), unmutated IgHV (gray, 3/5) and p53-deficient CLL (black, 8/9). Overall, these data suggest that targeting NEDD9-associated pathways such as the AURKA-HDAC6-CTTN signaling is a promising strategy for preventing CLL homing, thus limiting disease progression (Fig. 6F).

## Discussion

In this study we provide evidence that loss of the scaffold protein Nedd9 is sufficient to significantly delay CLL onset, impair leukemic infiltration, and prolong survival of the CLL mice. The effect of Nedd9 in promoting CLL progression was largely due to B-cell-intrinsic Nedd9 activity, Nedd9 deficiency led to impaired CLL cell adhesion and migration in vitro and mitigated tissue homing in vivo. NEDD9 levels in human peripheral blood CLL cells were associated with migration and adhesion signatures, and in human CLL lymph nodes NEDD9 was frequently expressed, either as diffuse pattern or remarkably accentuated in proliferation centers. Furthermore, NEDD9-depending CLL migration could be diminished by inhibition of effector kinases AURKA and HDAC6. Taken together, our study implies the potential of targeting CLL cell homing and CLL cell progression via NEDD9 signaling.

The role of NEDD9 in B-cell malignancies remains obscure to date, although the most apparent phenotype of Nedd9-deficient mice affects B-cells, with the marginal zone being almost empty of B-cells, and with lymphocytes being generally decreased in secondary lymphoid organs [16, 32]. Marginal zone B-cells, besides follicular B-cells, are discussed as the origin cells of CLL and the migration and chemotaxis processes enabling their homing to the immune niches are crucial for CLL progression [7]. Both the importance of NEDD9 for marginal zone B-cells and their migration capacity implies a role for NEDD9 in CLL pathogenesis [33].

NEDD9 has not been found mutated in CLL [34, 35] and heterogeneous expression of basal *NEDD9* mRNA has been reported in CLL cohorts in earlier studies [36, 37], similar to our observations. This is not surprising given the dynamic regulation of *NEDD9* mRNA and protein levels throughout the cell cycle and upon stimulation of cell surface receptors [13]. Furthermore, scaffolding proteins exert their functions depending on the cellular context based on posttranslational regulation such as specific phosphorylation patterns rather than quantitative RNA or protein expression.

Nedd9 depletion strongly delays CLL progression particularly at the progressive phase, which is independent of defects in leukemic cell proliferation or survival, but a consequence of hindered homing and migration. The fact that we did not observe more circulating cells in Nedd9-depleted mice suggests that CLL cells can only proliferate and recirculate after successful homing. Moreover, the enrichment of NEDD9 protein expression in CLL lymph nodes and in a subset of patients particularly in the proliferation centers also indicates an association between increased NEDD9 expression and successfully migrated CLL cells. In the proliferation centers, CLL cells receive essential signals for survival and proliferation including the BCR and NFκB activation [38, 39], and the success of BCR inhibition in CLL treatment has been proven to rely on their capacity to interfere with the CLL cell relocalization. The BCR can induce homing of CLL cells via activation of integrins. The VLA-4 heterodimer of Integrin-α4 (CD49d) and Integrin-β1 (CD29) was identified to be a robust prognostic marker with high expression of CD49d being associated with more aggressive disease and decreased survival [40–42]. Interestingly, activation of the BCR, chemokine receptors and ligation of β-integrins can lead to hyperphosphorylation of NEDD9 [13, 16]. Our results show that CLL cells exhibit impaired NEDD9 phosphorylation and lack of induction of *NEDD9* gene expression upon BCR stimulation compared to control B-cells, consistent with very rigid NEDD9 phosphorylation levels in human CLL cells compared to healthy B-cells. However, NEDD9 appeared to have no effect on the BCR-dependent activation of typical BCR effector kinases. Instead, CLL cells required Nedd9 to efficiently adhere to fibronectin or BMSCs, and to migrate towards CXCL12 or FBS, a mixture of stimulating factors. However, important receptors such as VLA-4- and CXCR4 - were not altered upon Nedd9 depletion, implying that Nedd9 regulates CLL cell motility via downstream signaling pathways.

In transformed cells, NEDD9 promotes their migratory capacity through cytoskeletal transformation and stabilizing membrane protrusions at leading edges [28]. The AURKA/HDAC signaling axis gets co-activated by NEDD9 upon external clues and has been shown to trigger CXCL12 dependent migration in transformed cells [29, 43]. Our observation that Nedd9-depleted CLL cells showed decreased level of CTTN together with the potency of AURKA and/or HDAC6 inhibitors to reduce CLL migration in a NEDD9-dependent manner strongly suggests a similar mode of action in CLL. Notwithstanding, inhibition of AURKA and HDAC6 or their combination could not fully recapitulate the migratory defect to the extent of Nedd9-knockout murine CLL cells, indicating that NEDD9 also has additional functions beyond the AURKA/HDAC6 signaling pathways.

Taken together, our results indicate NEDD9 as a promising target for CLL patients. Targeting a scaffolding protein without a kinase domain such as NEDD9 can be challenging although allosteric inhibition in general has been successfully demonstrated before [44].

Showing that specific inhibitors to AURKA and/or HDAC6 can mimic NEDD9 depletion advocates for further analyses of these therapeutic strategies in CLL. AURKA/B were found to be upregulated in bone marrow-derived CLL cells and AURKA inhibition is a promising target in preclinical models [45]. Interestingly, a recently completed phase I of alisertib in combination with the histone deacetylase inhibitor vorinostat in lymphoid malignancies presented encouraging results in diffuse large B-cell lymphoma (DLBCL) patients [46]. The HDAC6 inhibitor panobinostat also showed promising results in a phase II trial for relapsed DLBCL patients [47]. The independence of NEDD9 from the BCR (and BCL-2) encourages to further explore targeting the NEDD9 signaling axis as an alternative approach to prevent CLL homing, particularly in cases resistant to BCR inhibitors or venetoclax. It may be interesting to examine the efficacy of alisertib and panobinostat in combinations with targeted therapies such as ibrutinib or venetoclax in vivo to define their therapeutic potentials.

In summary, our study provides strong evidence for a functional role of NEDD9 in CLL pathogenesis that involves CLL cell intrinsic defects in cell adhesion and migration. These findings improve the understanding of CLL homing mechanisms, and may provide basis for the design of alternative therapeutic approaches by disrupting CLL migration to the protective niche.

## Supplementary information

Supplemental Information
