# Supplementary material for: The scaffold protein NEDD9 is necessary for leukemia-cell migration and disease progression in a mouse model of chronic lymphocytic leukemia
Source: Leukemia. 2022 May 6;36(7):1794–805. doi: 10.1038/s41375-022-01586-1 (PMC9252910; doi:10.1038/s41375-022-01586-1)
Supplement: Supplementary file 1 — Supplemental Information [file 41375_2022_1586_MOESM1_ESM.docx]

**Supplemental Information**

**The scaffold protein NEDD9 is necessary for leukemia-cell migration and disease progression in a mouse model of chronic lymphocytic leukemia**

*Running Title: NEDD9 promotes CLL cell migration*

Lisa Rusyn^1^, Sebastian Reinartz^1,2^, Anastasia Nikiforov^1^, Nelly Mikhael^1^, Alexander vom Stein^1,2^, Viktoria Kohlhas^1,2^, Johannes Bloehdorn^3^, Stephan Stilgenbauer^3^, Philipp Lohneis^4^, Reinhard Buettner^5^, Sandra Robrecht^1^, Kirsten Fischer^1^, Christian Pallasch^1,2^, Michael Hallek^1,2^, Phuong-Hien Nguyen^1,2*^ and Tamina Seeger-Nukpezah^1*^

*^*^P.-H.N. and T.S.-N. contributed equally to this study*

^1^ University of Cologne, Faculty of Medicine and Cologne University Hospital, Department I of Internal Medicine, Center for Integrated Oncology Aachen Bonn Cologne Duesseldorf, Germany.

^2^ CECAD Center of Excellence on Cellular Stress Responses in Aging-Associated Diseases, Center for Molecular Medicine Cologne, Germany.

^3^ Department of Internal Medicine III, Ulm University, Germany.

^4^ Hämatopathologie Lübeck, Reference Centre for Lymphnode Pathology and Haematopathology, Luebeck, Germany.

^5^ University of Cologne, Institute of Pathology, Germany.

**Correspondence**: Tamina Seeger-Nukpezah, University of Cologne, Department I of Internal Medicine, Weyertal 115c, 50931 Cologne, Germany; e-mail: [tamina.seeger-nukpezah@uk-koeln.de](mailto:tamina.seeger-nukpezah@uk-koeln.de); and Phuong-Hien Nguyen, University of Cologne, Department I of Internal Medicine, CECAD Research Center, Joseph-Stelzmann Str. 26, 50931 Cologne, Germany; e-mail: [hien.nguyen@uk-koeln.de](mailto:hien.nguyen@uk-koeln.de).

**Supplemental Data**

**
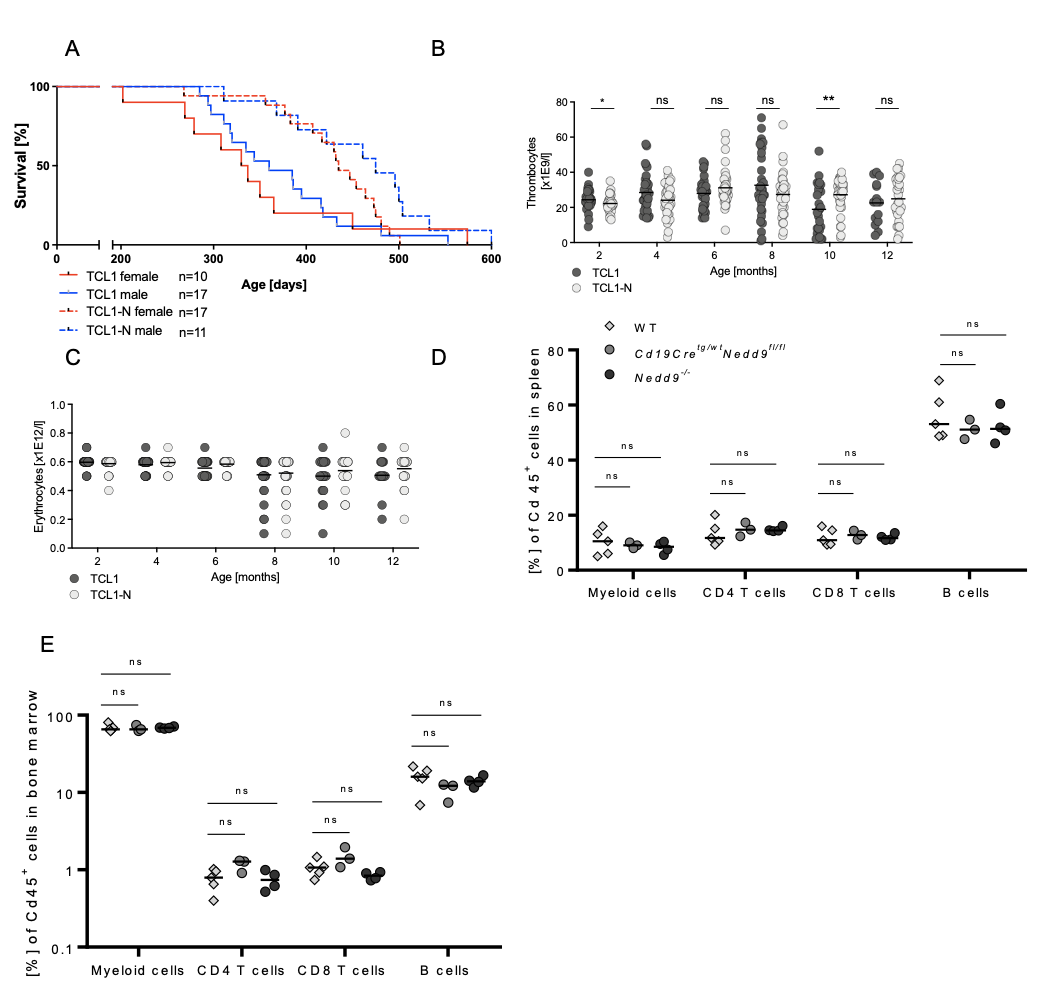
**

**
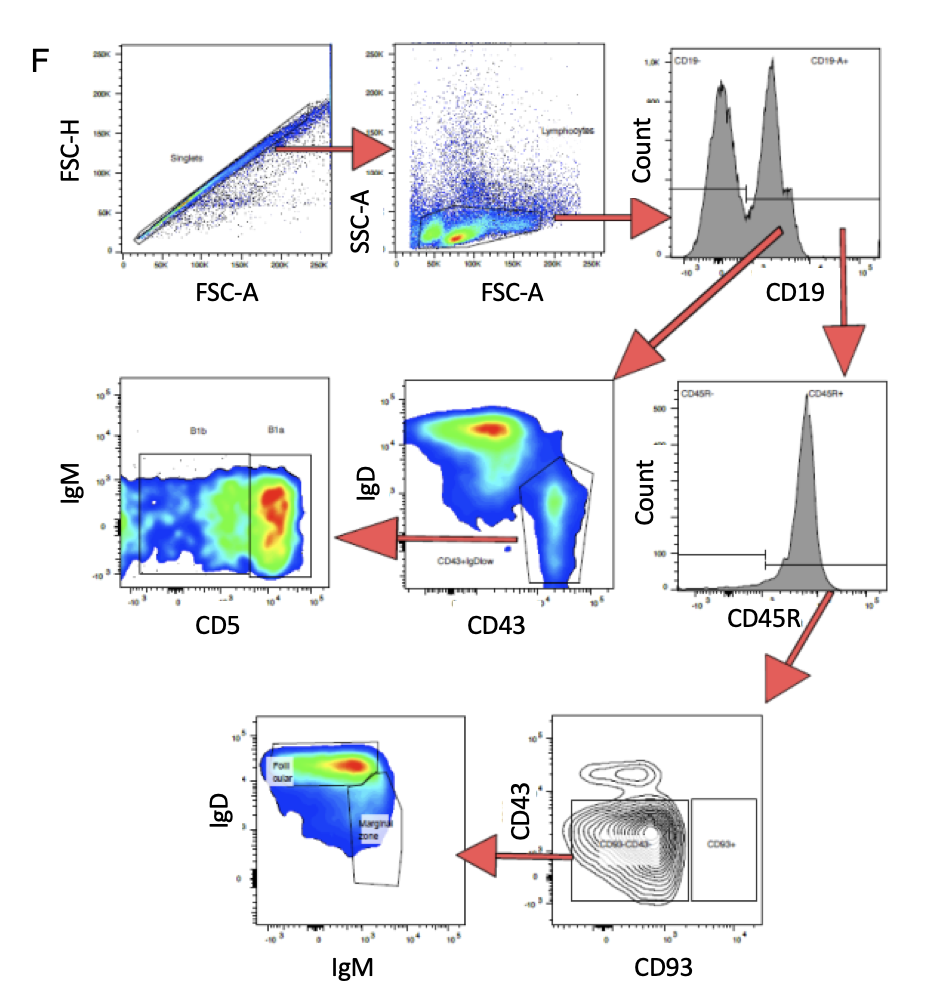
**

**Figure S1. Nedd9 depletion impairs CLL cell infiltration and prolongs survival in the Eµ-TCL1 mouse model.**

(A) Kaplan-Meier curve representing the overall survival of mice from birth to moribund of indicated groups.

(B) Thrombocyte count and (C) erythrocyte count of TCL1 and TCL1-N mice at indicated time points.

Analysis of indicated immune cells in (D) spleens and (E) bone marrow samples of WT, *CD19Cre^tg/wt^Nedd9^fl/fl^* and *Nedd9^-/-^* mice. Myeloid cells: CD11b^+^ ;CD4 T cells: CD3^+^CD4^+^; CD8 T cells: CD3^+^CD8^+^; B cells CD19^+^.

(F) Gating strategy to identify different B cell subsets including B1-a, B1-b, follicular and marginal zone in mouse spleen based on the expression of CD19, CD45R (B220), IgD, IgM, CD43, CD93.


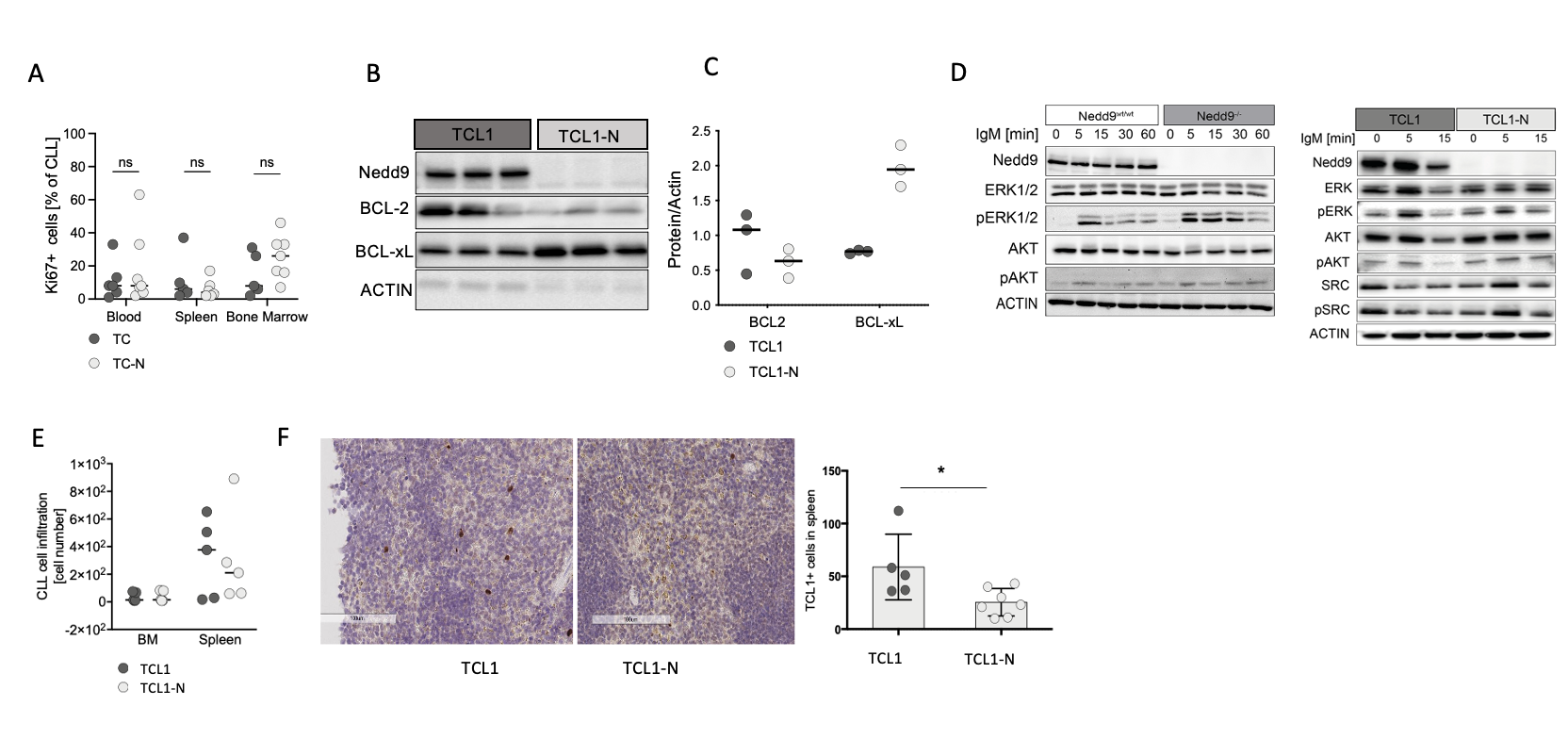


**Figure S2.** **Nedd9 loss impairs homing of CLL cells to lymphoid organs**.

(A) Flow cytometric analysis of CLL cell proliferation in blood, spleen and bone marrow samples of mice with (TCN) and without (TC) B lineage-specific Nedd9 loss at 10 months of age. P (blood) = 0.9184; p (spleen) = 0.5346; p (bone marrow) = 0.1578.

(B) CLL cells isolated from moribund TCL1 or TCL1-N mice and examined for protein levels of Nedd9, BCL-2, BCL-xL and β-actin by Western Blot with (C) quantification of BCL-2 and BCL-xL.

(D) Western blot analysis of B cells isolated from two different pools of 3 individual 3-month-old *Nedd9^wt/wt^* or *Nedd9^-/-^ mice*. BCR signaling was stimulated with 20µg/ml IgM (IgM F’ab fragment) for the indicated time points.

(E) Flow cytometric analysis of TCL1^+^ CLL cells homing to the spleen and bone marrow of C57Bl/6J mice 3 hours post intravenous injection. P (spleen) = 0.999; p (bone marrow) = 0.7460.

(F) Representative immunohistochemical staining of TCL1 from sections of spleens of recipient mice and quantification of TCL1 staining; p = 0.0186.


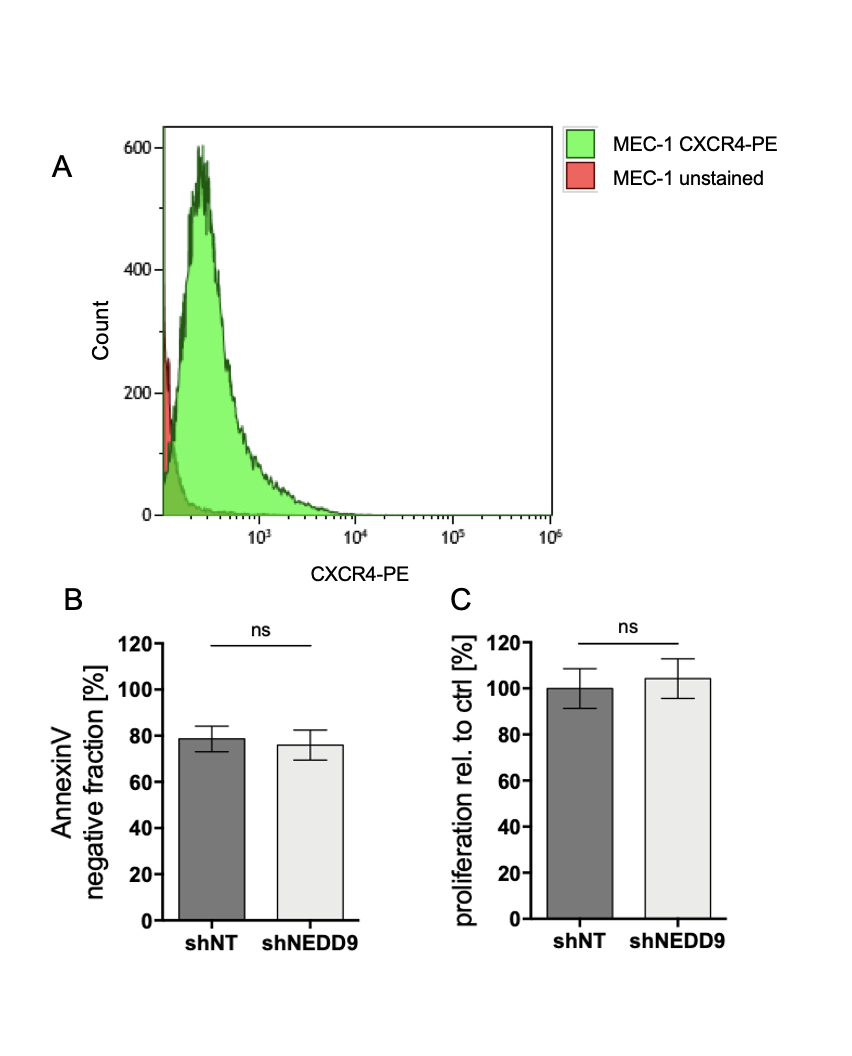


**Figure S3.** **Loss of Nedd9 has no impact on apoptosis or cell proliferation.** MEC1 cells were transfected with lentiviral vectors either expressing non-target shRNA (shNT) or shRNA directed to *NEDD9* (shNEDD9).

(A) Surface levels of CXCR-4 were analyzed by flow cytometry.

(B) Apoptosis was determined by Annexin-V flow cytometry; n = 6; p = 0.67.

(C) Proliferation was determined by BrdU incorporation relative to serum-free control; n = 6; p = 0.67. Statistics by Mann-Whitney test. Results represent mean ± SD.


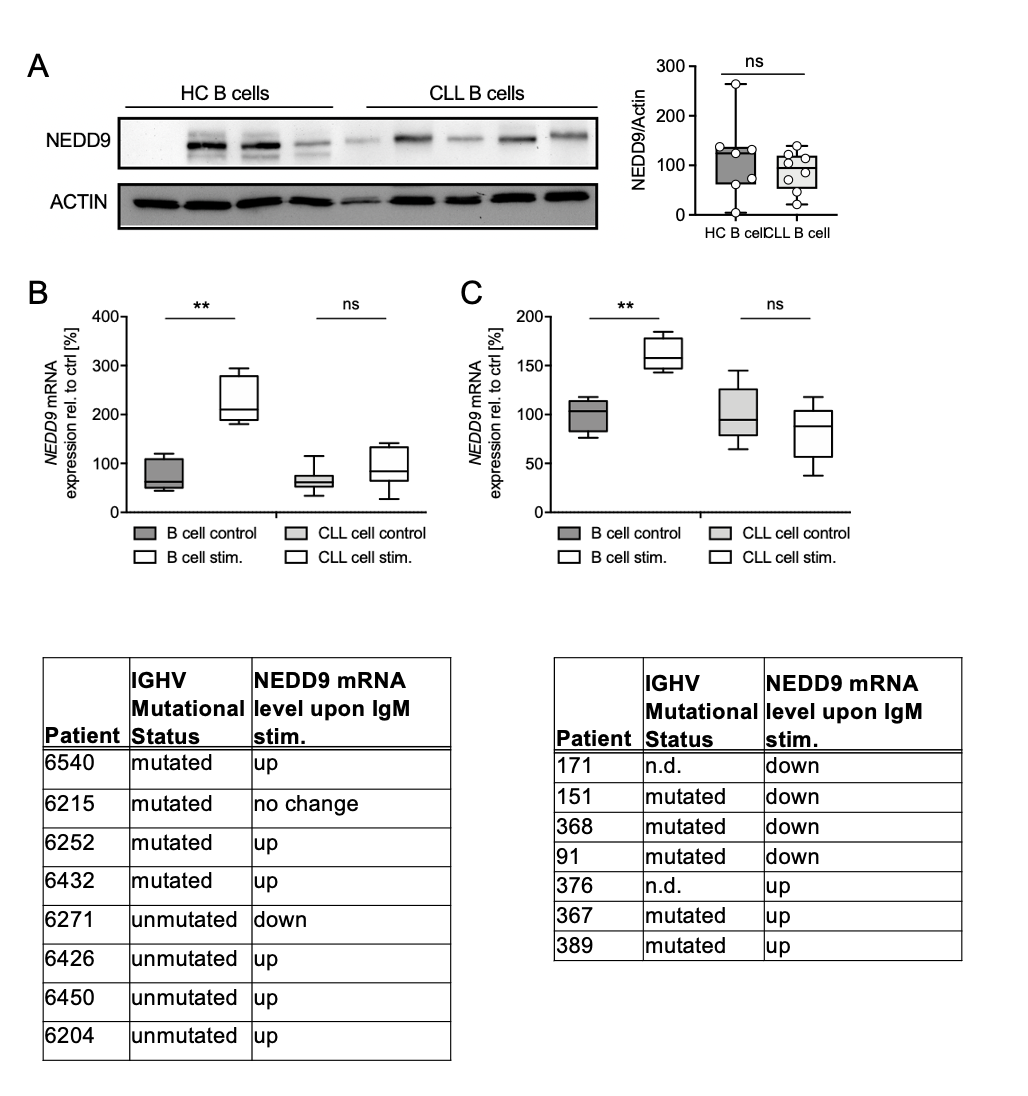


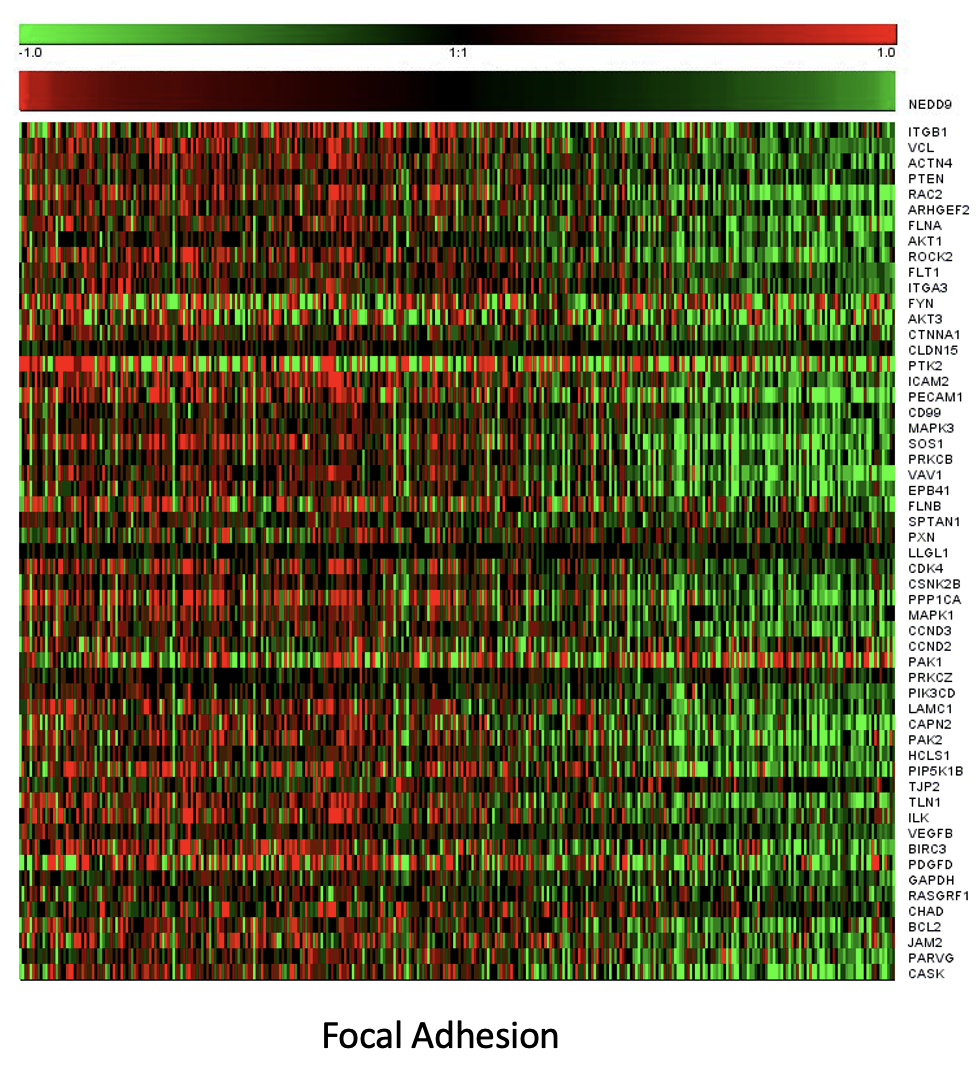


D


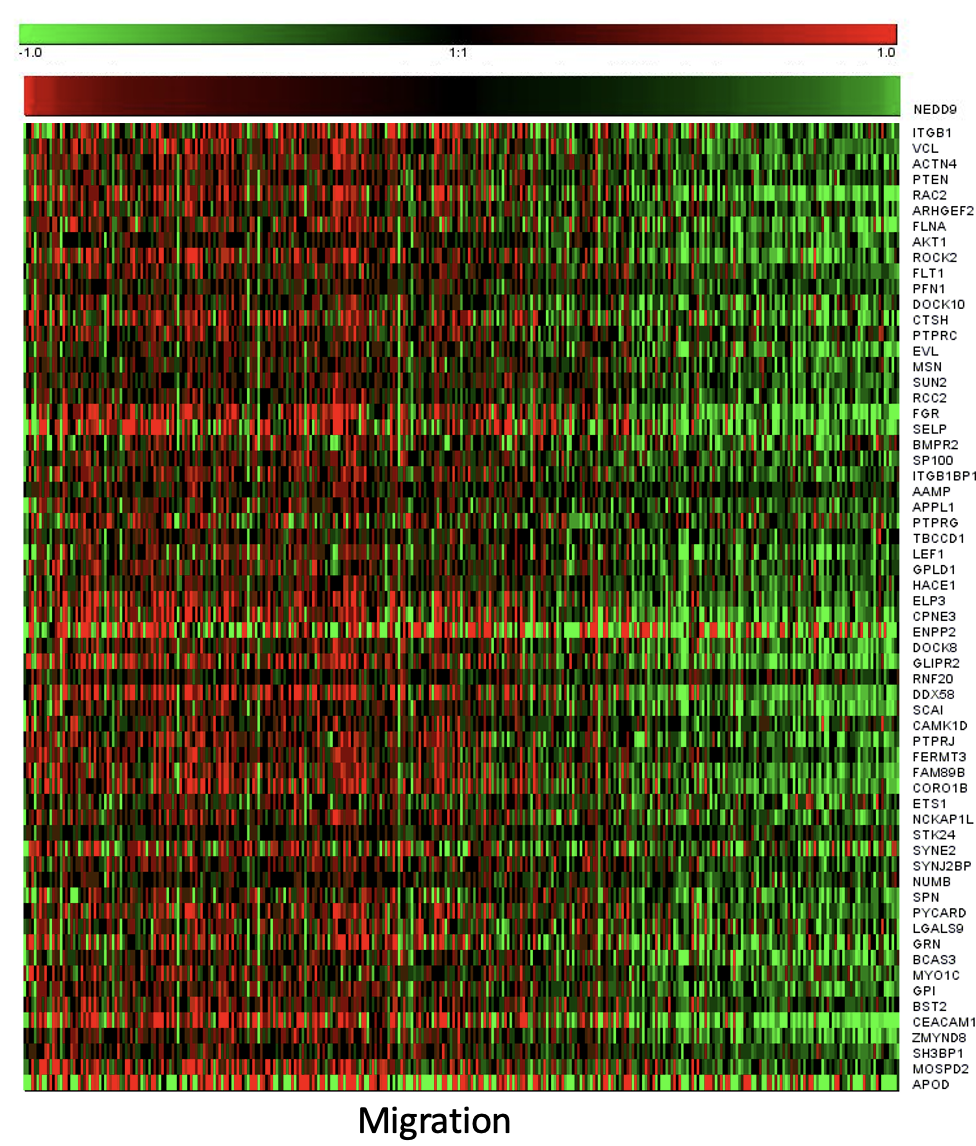


E


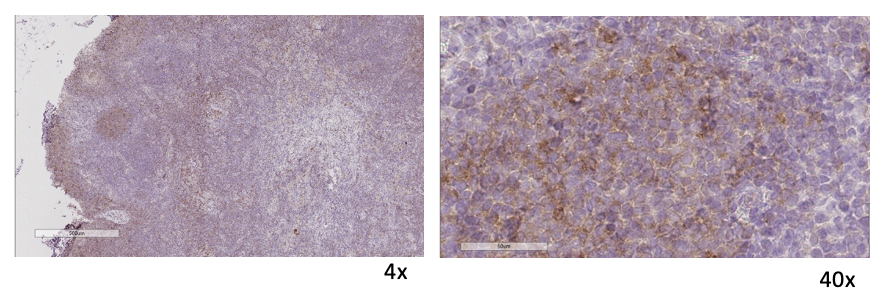


F

**Figure S4.** **NEDD9 upregulation upon BCR stimulation was impaired in CLL cells**.

(A) B lymphocytes were isolated from PBMC of CLL patients (CLL1-5) and healthy control donors (HC1-4) and NEDD9 level was analyzed by western blotting. Western blot quantification was analyzed by lab image 1D. Normalization on mean of all samples. n ≥ 8, p = 0.423.

(B) BCR signaling was stimulated with 20µg/ml IgM F’ab fragment. RNA was isolated 3 hours after stimulation followed by microarray analysis; p (B cell) = 0.0079, p (CLL cell) = 0.1026. The table below shows the IGHV status of the respective patient cohort.

(C) BCR signaling was stimulated with 20µg/ml IgM F’ab fragment. RNA was isolated 3 hours after stimulation and *NEDD9* level was analyzed using real-time PCR. n = 6, p (B cell) = 0.0022, p (CLL cell) = 0.3829. The table below shows the IGHV status of the respective patient cohort.

(D, E) Microarray analysis of expression levels of *NEDD9* as shown in Figure 5D-E but with naming of genes involved in (D) focal adhesions and (E) migration in CD19-sorted primary CLL samples (n = 337).

(F) Representative immunohistochemical staining of NEDD9 from sections of lymph node specimen from healthy control donors. Magnification 4x and 40x.


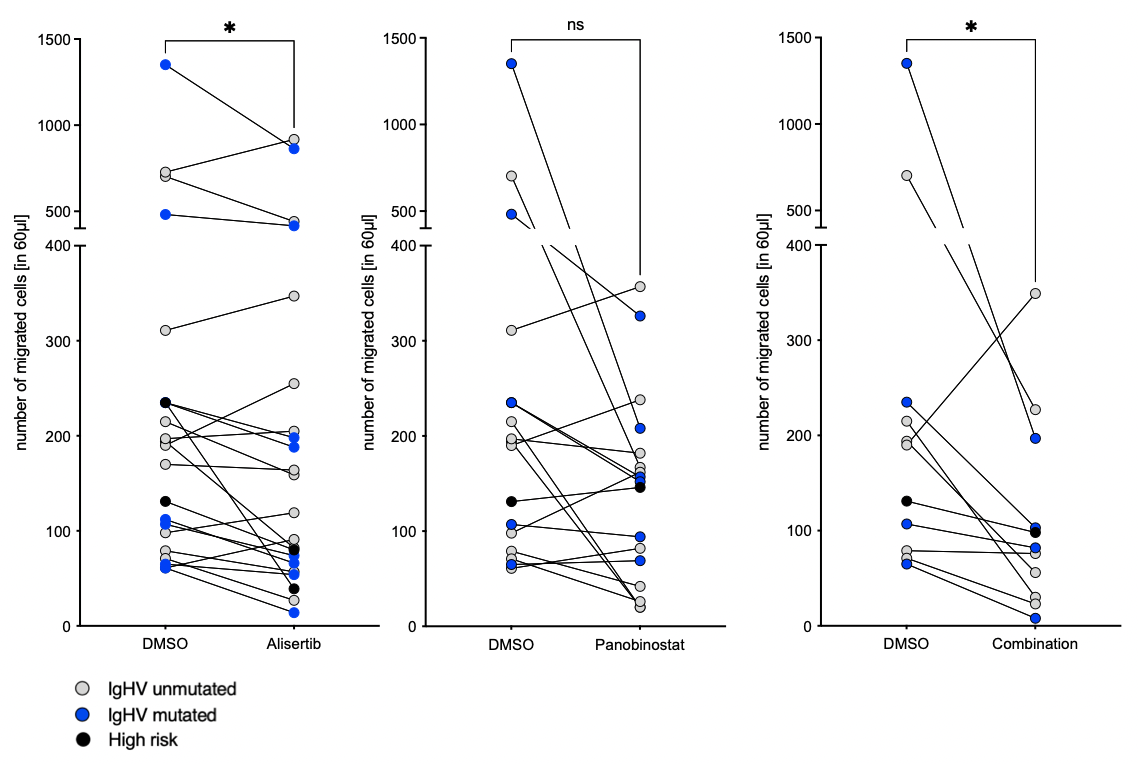


**Figure S5. Blocking of NEDD9-depending signaling axis reduces migration capacity of CLL cells.** Patient-derived primary CLL cells were treated with 0.5µM alisertib, 0.5µM panobinostat or a combination of both and examined for migration capacity towards 200ng/µl CXCL12 using a Boyden chamber. (A) Data represent the direct effect of alisertib treatment on number of migrated cells of individual patients of different CLL cell subgroups as indicated; p = 0.0186. (B) Data represent the direct effect of panobinostat treatment on number of migrated cells of individual patients of different CLL cell subgroups as indicated; p = 0.0585. (C) Data represent the direct effect of combination treatment on number of migrated cells of individual patients of different CLL cell subgroups as indicated; p = 0.0244. Statistics by Wilcoxon matched pairs test.

**Table S1. NEDD9 expression pattern in immunohistochemical NEDD9 stainings of lymph node sections from CLL patients.**

| **NEDD9 pattern** | **Cases** | **% of total** |
| --- | --- | --- |
| Negative | 6 | 28.57 |
| Diffuse | 8 | 38.10 |
| Focal | 7 | 33.33 |
| Total cases | 21 |  |

**Supplemental Experimental Procedures**

**Generation of the *Nedd9* conditional knockout mouse model**

The *Nedd9* gene (NCBI Reference Sequence: NM_001111324.2) is located on mouse chromosome 13. Eight exons have been identified, with the ATG start codon in exon 2 and the TGA stop codon in exon 8.

Exons 4~7 were selected as conditional knockout region. Deletion of this region results in the loss of function of the mouse Nedd9 gene. In the targeting vector, the Neo cassette was flanked by Rox sites. DTA was used for negative selection.

The constitutive KO allele will be obtained after Cre-mediated recombination. C57BL/6 ES cells were used for gene targeting. Mouse genomic fragments containing homology arms (HAs) and conditional knockout (cKO) region were amplified from BAC clone by using high fidelity Taq DNA polymerase, and were sequentially assembled into a targeting vector together with recombination sites and Neo Cassette as selection marker. The linearized vector was subsequently delivered to ES cells (C57BL/6) via electroporation, followed by drug selection, PCR screening, and Southern Blot confirmation.

**Antibodies and Reagents**

**For Western Blot**, primary antibodies specific for AKT (9272), BCL-xL (2764), ERK1/2 (9102), NEDD9 (4044), phospho-p130Cas (4011), phospho AKT (9271), phosphor ERK1/2 (9101) were purchased from Cell Signaling Technology (Berverly, MA). Primary antibody for BCL-2 (sc-7383) was purchased from Santa Cruz Biotechnology (Heidelberg, Germany). Primary antibodies for actin (ab49900) and cortactin (ab81208) were obtained from Abcam (Cambridge, England). Antibody for acetyl-cortactin (09-881) was obtained from Sigma-Aldrich. HRP-coupled secondary antibodies goat anti mouse IgG (70761) and goat anti rabbit IgG (7074) were purchased from Cell Signaling Technology. **For flow cytometry**, fluorochrome conjugated antibodies for CD19 (115523), CD20 (302319), CD3 (100204 and 300317), CD5 (100607 and 45005182), CXCR4 (146505), CD44 (103008), VLA-4 (103605), CXCR2 (149603), Ki67 (652403), CD11b (101208), CD4 (100414), CD21/35 (123407), IgD (405706), CD93 (136511), CD43 (143209), CD45R (103224) were obtained from Biolegend (San Diego, CA). Antibodies for CD3 (130-102-314), CD8 (130-122-017), CD5 (130-122-704), CD19 (130-118-463) and CD45 (130-102-412 and 130-110-664) were purchased from Miltenyi Biotec (Bergisch Gladbach, Germany). Antibody for IgM (550676) was purchased from BD Biosciences. **For immunohistochemistry** primary antibody specific for NEDD9 (4044) was purchased from Cell Signaling Technology (Berverly, MA). Primary antibody for TCL1 (ab108978) was obtained from Abcam (Cambridge, England). Primary antibody for Ki67 (275R-16) was purchased from Cell Marque (Rocklin, CA).

**Western blotting**

Samples and standards were separated at 120V for 60-100 minutes using the Mini-PROTEAN® Tetra Cell Systems (Biorad, Hercules, CA), followed by transfer to a Polyvinylidene difluoride (PVDF) membrane at 25V for 40 minutes. Membranes were incubated with respective antibodies as described in the supplemental information. Signal intensity levels were compared based on densitometry analysis using the Lab Image 1D software (Kapelan Bio-Imaging GmbH, Leipzig, Germany).

**Flow cytometry**

Labeling of intracellular proteins was performed using IntraPrep Permeabilization Reagent (A07803, Beckman Coulter) according to manufacturer’s protocol. For the determination of apoptotic cells FITC Annexin V kit (Biolegend) was used according Manufacturer’s instructions. Measurements were performed on FACSCalibur (Becton Dickinson, Franklin Lakes, New Jersey, United States) or Gallios (Beckman Coulter, Brea, California, United States) cytometers. Data storage and processing was done using the KALUZA (Beckman Coulter, Brea, California, United States) software packages.

**Murine sample purification and cell culture**

Primary murine CLL cells were isolated from spleens of leukemic mice by density gradient centrifugation. The final purified cell population contained more than 80% of CD19^+^ CD5^+^ lymphocytes. Murine CD19^+^ B cells were purified from crude spleen suspension after erythrocyte lysis with ACK buffer using mouse CD19 MicroBeads according to the manufacturer’s protocol. Murine CLL and B cells were cultured in RPMI supplemented with 10% FBS.

**Human sample purification and cell culture**

Malignant B cells were isolated from CLL patients treated at the Department of Internal Medicine I of Cologne University Hospital harboring a peripheral blood lymphocyte count of ≥ 25 × 10^9^ L^-1^. Age and sex-matched healthy donors were included as control. CLL B cells were separated (> 95%) using Ficoll sucrose gradient and further enriched by depletion of non-B cells by the RosetteSep Human B cell enrichment cocktail. Healthy-donor PBMC were isolated by density gradient centrifugation and CD19^+^ B-cell fraction was purified by CD19 MicroBeads according to the manufacturer’s instructions. CLL and B cells were cultured in RPMI + 10% heat inactivated FBS. Cells were maintained in a humidified 5% CO_2_ atmosphere at 37°C in a BINDER incubator (BINDER GmbH). Cells were serum starved for 3 hours before stimulation with in-solution 20 µg/ml goat anti–human immunoglobulin M (IgM) antigen-binding fragment for the indicated times.

**Lentivirus production and infection of cells**

To generate infectious lentiviral particles, HEK395T cells (DSMZ No. ACC 635) were co-transfected with helper plasmids and constructs encoding shRNA sequences either against NEDD9 (shNEDD9 sequence: CCTCCTTCTCATACCACTCAA) or no target (shNT sequence: CAACAAGATGAAGAGCACCAA) using the calcium phosphate (CaPO_4_) coprecipitation technique (Carlotti et al., 2004). Virus titer was quantified using HIV 1 p24 antigen enzyme-linked immunosorbent assay (ELISA) kit. To generate MEC1 (DSMZ No. ACC 497) cells stably expressing either shNEDD9 or shNT, cells were infected with a multiplicity of infection (MOI) of 5 in the presence of 2µg/ml polybrene followed by a centrifugation step of 45 min at 300x *g.* Since both constructs contain a puromycin resistance gene, selection was started 24 hours after transduction, where medium was changed to growth medium supplemented with 2µg/ml puromycin for the selection of transduced cells. Selection pressure was maintained over 12 to 15 days with a medium change every 2-3 days. A single cell dilution was performed for both cell lines (MEC1 shNT and MEC1 shNEDD9) to generate monoclonal cultures that were further used for the experiments.

**Cell proliferation assay**

Incorporation of Bromodeoxyuridine (BrdU) into newly synthesized DNA of dividing cells was determined by the colorimetric Cell Proliferation ELISA kit (11647229001, Roche, Basel, Switzerland), according to the manufacturer's protocol. 1x10^5^ serum-starved cells were seeded in a 96-well plate in 10% FCS-containing RPMI and starvation medium as control before BrdU was added for 4 hours. Incubation with peroxidase-conjugated anti-BrdU was performed for 60 minutes, and measurement of absorbance was carried out after 5-10 minutes using the PARADIGM plate reader (Beckman Coulter).

**RNA isolation, reverse transcription and qPCR**

RNA was isolated using RNeasy® Mini Kit followed by reverse transcription was done using GoScript™ Reverse Transcription System according to the manufacturer’s protocol. Quantitative real-time PCR for NEDD9 was run on a 7500 Fast cycler system (Applied Biosystems, Foster City, California, United States) using GoTaq® qPCR Master Mix. mRNA levels were normalized to deltaCT geometric mean of β-actin and TATA box binding protein (TBP).

**List of abbreviations**

| AURKA | Aurora kinase A | |
| --- | --- | --- |
| BCR | B cell receptor | |
| CFSE | Carboxyfluorescein succinimidyl ester | |
| CLL | Chronic lymphocytic leukemia | |
| CTTN | Cortactin | |
| ERK1/2 | extracellular signal-regulated kinase | |
| FAK | Focal adhesion kinase | |
| FN | Fibronectin |  |
| HDAC6 | Histone deacetylase 6 | |
| NEDD9 | Neural precursor cell expressed, developmentally down-regulated 9 | |
| NSG | NOD-*scid* IL2Rg^null^ | |
| SRC | Proto-oncogene tyrosine-protein kinase Src or sarcoma | |
| TCL1 | T-cell leukemia/lymphoma protein 1A | |
| TME | Tumor microenvironment | |
| VLA-4 | Very Late Antigen-4 | |
